# The effectiveness of platelet-rich plasma on the skin wound healing process: A comparative experimental study in sheep

**DOI:** 10.14202/vetworld.2018.800-808

**Published:** 2018-06-14

**Authors:** Daikh Badis, Bennoune Omar

**Affiliations:** 1 Department of Biology of Organisms, University of Batna 2, Batna, Algeria; 2 Biotechnology’s Laboratory of the Bioactive Molecules and the Cellular Physiopathology, University of Batna 2, Batna, Algeria; 3 Department of Veterinary Science, Institute of Agronomic and Veterinary Sciences, University of Batna 1, Batna, Algeria

**Keywords:** healing, platelet-rich plasma, sheep, skin

## Abstract

**Aim:**

The therapeutic evaluation of the biological effect of platelet-rich plasma (PRP) used as a surgical adjunct to maintain the inflammatory process and to potentiate tissue healing, make the subject of recent research in regenerative medicine. This study was designed to evaluate the healing activity of PRP by its topical application on the skin experimentally injured in a sheep model.

**Materials and Methods:**

The study was conducted on 9 adult and clinically healthy males sheep. PRP was obtained by a protocol of double centrifugation of whole blood from each animal. After sterile skin preparation, full-thickness excisional wounds (20 mm x 20 mm) were created on the back of each animal. The animals were randomly divided into three equal groups of three sheep for each. In Group I, the wounds were treated with PRP, in Group II; wounds were treated with Asiaticoside; in Group III, wounds were treated with saline solution. The different treatments were administered topically every 3 days. Morphometric measurements of the contraction surface of the wounds and histopathological biopsies were carried out at the 3^rd^, 7^th^, 14^th^, 21^st^, and 28^th^ days of healing.

**Results:**

The results of the morphometric data obtained revealed that it was significant differences recorded at the 7^th^ and 14^th^ day of healing in favor for animals of Group I. Semi-quantitative histopathological evaluation showed that PRP reduces inflammation during 3 first days post-surgical and promotes epithelialization in 3 weeks of healing.

**Conclusion:**

We concluded that topical administration of PRP obtained by double centrifugation protocol could potentially improve the skin healing process in sheep.

## Introduction

The development of bioactive surgical adjuvant capable of maintaining the inflammatory process and potentiating healing is one of the great challenges of regenerative medicine. Indeed, all medical disciplines calling for tissue self-repair techniques remain a recurring problem and object of discussion of innovative research [1]. The autologous platelet concentrate from the fibrin glue technology of the 1990s offers a new therapeutic strategy and a challenge of hope at the center of attention of clinicians, especially against refractory pathologies and rebels to conventional treatments, as well as it can be expensive, easy, and quick to manufacture. Moreover, since January 2011, it is no longer included on the list of doping products [2]. In this research perspective, several studies have been conducted but widely controversial, the main divergence of which is the absence of a consensus that normalizes a standard protocol for the preparation of platelet-rich plasma (PRP), with evaluation of the biological effects associated with its administration [3,4].

Wound healing is one of the most complex biological events after delivery [5]. It is a physiological process that enables tissue restoration through an organized cascade of complex cellular and molecular events. Classically, there are two ways of the healing skin wounds following the conditions of trauma and treatment. Healing by primary intention, wounds heal quickly and without contraction, and wounds with loss of substance heals less quickly or by the second intention. Among the biological events that generate healing, the inflammatory step, sometimes called even crucial, often initiated by a mechanism of hemostasis which aims to prevent the accidental loss of blood. Ostvar *et al*. [6] reported that platelets play a vital role in the healing process of cutaneous wounds, and their activations are accompanied by the secretory production of the various growth factors that act either directly or indirectly on all aspects of the healing cascade.

Healing by second intention is prone to skin complications in sheep. In veterinary practice, the increased demand for products that accelerate healing and improve the esthetic appearance is the subject of several medical and economic researches. The objective of this study was to evaluate the healing activity of PRP by its topical application to experimentally injured skin in sheep.

## Masterials and Methods

### Ethical approval

All maneuvers and surgical procedures were performed in accordance with the conditions established by the Ethics Committee of the Institute of Agronomic and Veterinary Sciences of the University of Batna 1 - Algeria.

### Experimental animals

A total of nine clinically healthy male sheep, weighing 20-25 kg and aged 6 months were used in this study, raised in the animal facility of the Institute of Veterinary and Agricultural Sciences - Batna 1 - during an adaptation period of 15 days and a trial period of 28 days. All these animals had free access to water and the regular diet (straw and barley). 1 month prior to injury, all sheep were dewormed with ivermectin at a dose of 0.2 ml/kg administered subcutaneously.

### Preparation of PRP

A volume of 20 ml of whole blood was collected from each sheep by septic puncture of the jugular vein. This sampling requires the use of two tubes of 15 ml capacity containing 2.25 ml of citrate acid dextrose (ACD) for each tube, filled with blood up to graduation 10. The blood collected was transferred to empty tubes of 5 ml capacity using a 1 ml pipette. A total of 5 tubes containing 4 ml of blood for each, which have undergone centrifugation. A modified double centrifugation protocol proposed by Carneiro *et al*. [7] was used in this study, performed by a laboratory centrifuge (Hettichzentrtrifugen D-78532 Tuttlingen). The speed of the 1^st^ centrifugation was set at 1800 r.p.m for 8 min resulting in two basic components: Components of the blood cells in the lower fraction and the serum which corresponds to the upper fraction. All contents above the red blood cells were pipetted and transferred to another empty tube of 5 ml capacity without anticoagulant. These samples were again centrifuged at 1000 r.p.m for 8 min. It results a small red fraction at the bottom of each tube and a clear supernatant at the top. A volume of 0.5 ml of the clear supernatant above the red fraction which was pipetted corresponds to the PRP. The total amount of PRP collected is 2.5 ml. All these steps of preparing the PRP were carried out at room temperature (22°C).

### Platelets count

The count of platelets was manually performed using a hemocytometer (Neubauer cell improved).

### Realization of smears

The purpose of performing whole blood smears and PRP (MGG staining) is to analyze platelet richness and to assess the efficacy of dextrose (ACD) as an anticoagulant in sheep. All smears were performed at each count.

### Technical of the injury

Before all surgical procedures, the animals were first tranquilized with acepromazine at a dose of 0.1 mg/kg intramuscularly. After then, the skin was prepared for aseptic surgical procedure by application of an iodine solution of povidone (10% dermal betadine). On the back of each animal, four complete excision wounds of the skin (20×20 mm) were performed on each lateral side and in the vicinity of the vertebral line. Excision was performed under local anesthesia (lidocaine hydrochloride 2%), induced by subcutaneous infiltration at a flow rate of 1 ml/1 cm^3^ [8]. All the rules of asepsis were respected, and all the wounds remain open and not sutured. The animals were randomly divided into three equal groups of three sheep for each. The administration of the various treatments was performed topically once every 3 days, and the PRP was prepared for each application and activated in addition of chlorure of calcium (0,1 ml of CaCl_2_ to each 1 ml of PRP) immediately before its administration [9].

- Group I: Animals treated with PRP.
- Group II: Animals treated with asiaticoside at 2%.
- Group III: Animals treated with saline solution (NaCl 9%).

### Post-operative follow-up

A general clinical examination was performed in all animals (animal behavior, body temperature, and cardiorespiratory activity). Within the wounds, a daily macroscopic follow-up was carried out during the whole period of experimentation.

### Healing follow-up

#### Percentage of contraction of the wound

The contraction surface of the wound was measured using a digital caliper on the 3^rd^, 7^th^, 14^th^, 21^st^, and 28^th^ postoperative days until the wounds were healed. The average percentage of contraction of the wound was calculated according to the equation established by Singh and Sharma [10].

The percentage of wound contraction = 100 × (wound surface on 0^th^ day - wound surface on n^th^ day)/Wound surface on day 0; where n = number of days 3^rd^, 7^th^, 14^th^, 21^th^, and 28^th^ day.

#### Semi-quantitative histopathological evaluation

Samples of the skin using a biopsy punch from lesion sites of excisions were performed on the 3^rd^, 7^th^, 14^th^, 21^st^, and 28^th^ postoperative days. The samples were processed by routine histological procedures, first fixed with buffered formaldehyde, then embedded in a paraffin solution, and cut transversely by a microtome into 4 μm thin sections. Tissue sections were stained with hematoxylin-eosin and examined for possible histopathological changes. The histopathological scores established by Vidinsky *et al*. [11], were used in this study, presented as codes from 0 to 3 (Table-1).

**Table-1:** Description of the differents histopathological scores of the semi-quantitative evaluation.

Score	Epithelialization	PMNL	Macrophages	Fibroblasts	Neo-angiogenesis	Neo-collagen
0	Thickness of cut edges	Minimum	Minimum	Minimum	Minimum	Minimum
1	Migration of epithelial cells	mild	mild	mild	mild	mild
2	Bridging of the excision	Moderate	Moderate	Moderate	Moderate	Moderate
3	completeregeneration	Marked	Marked	Marked	Marked	Marked

PMNL=Polymorphonuclear leukocyte

### Statistical analysis

Descriptive statistical analysis was performed for each variable in this study. The analysis of variance ANOVA between the different groups followed by the least significant difference test, allows specifying the variations of the percentage of contraction. The nonparametric U-test of Mann-Whitney was used for a statistical analysis of the different histopathological scores obtained. SPSS software 23.0 (2015) was used for all analyzes. The values of p<0.05 and p<0.01 were used to determine the level of significance of the differences recorded.

## Results

### 

#### Plasma rich in platelets

**Figure-1:**
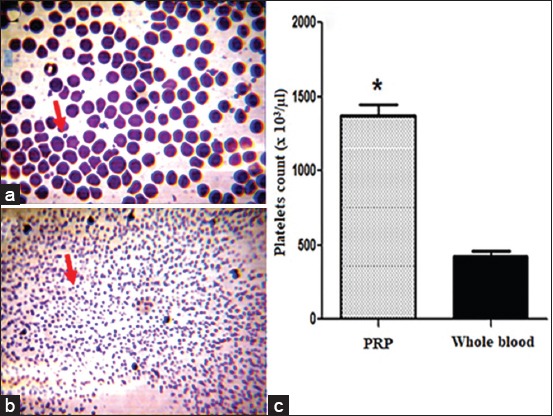
(a) Smear showing platelets (arrows) of whole blood. (b) Smear showing platelets (arrows) of platelet-rich plasma (PRP) (M.G. G×100 stain). (c) Average number of platelets in whole blood and in PRP. *Denotes statistically different significance compared with whole blood (p<0.05)

The results of the manual counting of thrombocytes using a hemocytometer revealed that the average number of platelets obtained is much higher in the PRP (1374.8±164.8.92×10^3^/μl) in comparison with whole blood (425.26±75.23×10^3^/μl). It is approximately thrice higher than that of whole blood (Figure-1c). Repeated observations of the different smears performed, revealed the complete absence of the platelet aggregation phenomenon (Figure-1b), and thus confirmed the importance of the use of dextrose (ACD), as an anticoagulant of choice during the preparation of PRP in the sheep.

#### Clinical follow-up

No changes were observed in the behavior of the animals. All animals well tolerated this procedure, and all sheep do not seem affected by the presence of wounds on their backs. No symptoms of aggression or hypersensitivity were observed during the application of the different treatments.

#### Macroscopic follow-up of wounds

**Figure-2:**
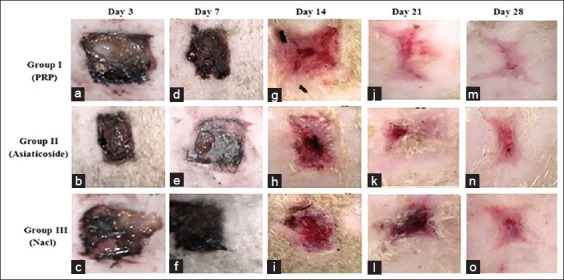
(a-o) Macroscopic observations of healing at different periods of time.

Within different wounds (Figure-2), no complication was observed in lesional sites, such as purulent infections or exuberant granulation tissue formation. However, careful macroscopic observation of different excision wounds has shown progress in contraction of wounds over time. At the end of the 14^th^ day, the reduction of the surface of wounds was observed remarkably (Figure-2g) in wounds for animals of Group I (wounds treated by topical application of PRP) in comparison with animals of Groups II and III (wounds treated with Asiaticoside (Figure-2h) and saline solution (Figure-2i), respectively).

#### The parameters of healing

Contraction surface of the wound

**Figure-3:**
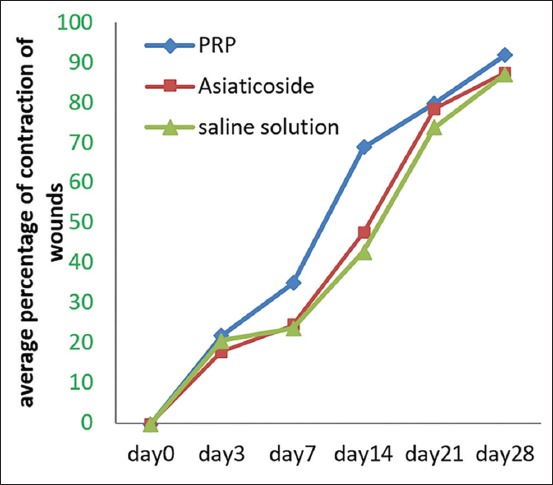
Variations in the percentage of contraction of the wound according to the different treatments administered.

The results represented by curves in Figure-3 showed the progress of wound contraction in all animals. This progress is particularly remarked in wounds of animals of Group I. At equal rank, careful examination of the results listed in Table-2 revealed significant differences between the different groups of animals in this study. After 1 week postsurgical, the average rate of contraction of wounds in Group (I) was statistically significant (p<0.05) compared with those in Groups (II) and (III). We note that the mean percentage of contraction in the group of animals treated with PRP was (34.93±6.11) whereas it was (24.43±2.96) and (23.64±8.63) in the group of animals treated with asiaticoside and the saline solution, respectively. After 2 weeks of injury, the difference is highly significant (p<0.01) between the different groups. The mean percentage of wound contraction for animals of Group I treated with PRP was (68.80±4.64) compared with (47.66±5.09) and (42.90±9.38) in Groups II and III, treated with asiaticoside and saline solution, respectively.

**Table-2:** Variations of the percentage of contraction of the wound in the different groups.

Day of observation	Groups	Mean±SD	n	95% CI		F	p

				Lower limit	Upper limit		
Day 3	I	21.86±3.70	12	12.65	31.06	0.764	0,506
	II	17.95±4.19	12	7.52	28.38		
	III	20.78±4.07	12	10.66	30.90		
Day 7	I	34.93±6.11*	12	25.20	44.65	4.001	0.057*
	II	24.43±2.96*	12	20.14	28.71		
	III	23.64±8.63*	12	9.90	37.37		
Day 14	I	68.80±4.64**	12	57.26	80.33	13.737	0.004**
	II	47.66±5.09**	12	39.55	55.77		
	III	42.90±9.38**	12	19.58	66.22		
Day 21	I	79.83±1.45	12	76.22	83.43	2.935	0.119
	II	78.55±3.78	12	75.53	84.57		
	III	73.71±3.80	12	94.25	83.16		
Day 28	I	91.90±1.63	12	87.85	95.95	0.345	0.718
	II	87.55±9.78	12	71.97	103.12		
	III	87.17±8.79	12	73.18	101.16		

Group I=Animals treated with PRP; Group II=Animals treated with asiaticoside at 2% and Group III=Animals treated with saline solution (NaCl 9%). M=Mean, SD=Standard-deviation, CI=Confidence interval,

* Denote a significant difference (p<0.05),

** Denote a highly significant difference (p<0.01)

### Semi-quantitative histopathological evaluation

**Figure-4:**
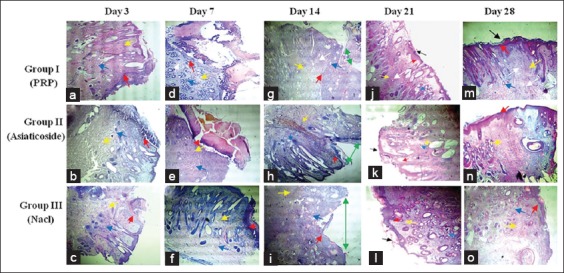
Micrograph of various histological aspects (transverse section) from cutaneous biopsies performed in the 3^rd^ (a-c), 7^th^ (d-f), 14^th^ (g-i), 21st (j-l) and 28^th^ (m-o) day after injury (The magnification is 40×). Red arrow (re-epithelialization), blue arrow (neovascularization), yellow arrow (newly formed fibrin network), black arrow (keratinization), green arrow (injury site) and white arrow (newly formed hair follicle). At day 3, epithelialization was observed on the upper part of the inner wall of the wound margins in the different groups, well marked in wounds treated with platelet-rich plasma (PRP) and asiaticoside (a and b). The edges of the wound were covered by blood coagulum and inflammatory cells in the control wounds (c). Nothing is particular about the other parameters. At day 7, accentuation of epithelialization in wounds treated with PRP, the epithelium appears to cover all the free wound edge (d). No connection of the wound margins in the different groups. A large unorganized collagen deposit was observed in wounds treated with PRP (d), random loose collagen bundles observed in the other groups (e and f). For other parameters, neovascularization is important in the group treated with PRP (d) and appearance of hair follicles. Persistence of inflammatory infiltrate in control wounds (f). At day 14, union of the wound margins in the different groups (g-i), almost complete with a better developed granulation tissue in wounds treated with PRP (g). At day 21, the new collagen bundles densely filled the junction of the wound into the PRP (j) treated group. At day 28, complete healing in different groups (m-o) with different aspects, complete keratinization of the skin, perfect reorganization of collagen and reappearance of hair follicles in wounds treated with PRP (m), advanced fibrosis in treated group by asiaticoside (n) and poor delayed healing in the control group (o).

**Figure-5:**
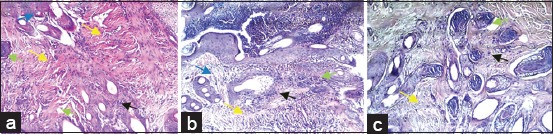
Hematoxylin and eosin stains of wound tissue at day 3 (a-c) after injury. (The magnification is ×100). Blue arrow (neovascularization), yellow arrow (newly formed fibrin network), black arrow (nuclear polymorphic leucocytes) and green arrow (macrophage). (a): Section of a wound treated by platelet-rich plasma, massive recruitment of inflammatory cells, large collagen deposition and a reduced number of macrophages. (b) Representative tissue from wounds treated with asiaticoside, moderate inflammatory cells, and a looser collagen network with a small number of macrophages. (c) Tissue from wounds treated with saline solution, has a much reduced number of inflammatory cells and an important number of macrophages.

**Figure-6:**
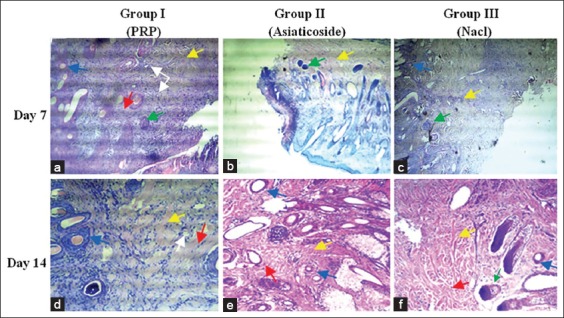
Hematoxylin-eosin stains from wound tissue made at day 7 (a-c) and 14 (d-f) after injury. (The magnification is 100×). Yellow arrow (newly formed fibrin network), red arrow (Fibroblasts), blue arrow (neovascularization), white arrow (hair follicle) and green arrow (macrophage). At day 7, a significant number of fibroblasts, and newly formed collagen bundles observed in wounds treated with platelet-rich plasma (PRP) (a), which the bundles show fibers left sparse, and that they appear to not fill the wound site. In the other groups, collagen is less abundant with irregular appearance and persistence of macrophages. At day 14, numerous fibroblasts observed in wounds treated with PRP (d), which piles up between condensed bundles of collagen. Neovascularization is important with reappearance of hair follicles. In the other groups, loose, unorganized collagen was observed (e and f).

Histopathological examination (Figures 4-6) and semi-quantitative evaluation (Table-3) showed differences in the progression of healing in the different groups.

**Table-3:** Results of the semi-quantitative histopathological evaluation. Each calculated parameter was presented by its mean and standard deviation (mean±SD).

Jour	Group	Epithelization	PMNL	Macrophages	Fibroblasts	Neo-angiogenesis	Neo-collagen
Day 3	I	0.9±0.73	2.4±0.48*	1.3±0.48	1.6±0.51	1.4±0.51	0.7±0.48
	II	0.5±0.52	1.9±0.73	1.4±0.51	1.6±0.51	1.4±0.51	0.4±0.51
	III	0.3±0.48	1.5±0.7	1.5±0.48	1.4±0.51	1.2±0.42	0.3±0.48
Day 7	I	2.2±0.78	0.9±0.73	1.2±0.42*	2.1±0.73*	1.63±0.5	1.6±0.51
	II	2.1±0.73	0.8±0.63	1.5±0.51	1.7±0.48	1.5±0.52	1.4±0.51
	III	1.8±0.91	0.7±0.67	1.6±0.91	1.6±0.51	1.3±0.48	1.3±0.48
Day14	I	2.8±0.42	0.6±0.52	1.0±0.52	2.7±0.48*	2.6±0.51	2.7±0.48
	II	2.6±0.51	0.5±0.52	1.2±0.51	2.5±0.52	2.3±0.48	2.5±0.52
	III	2.4±0.51	0.3±0.48	1.3±0.61	2.4±0.51	2.1±0.73	2.4±0.51
Day 21	I	3.0±0*	0.5±0.52	0.5±0.52	3.0±0	3.0±0	2.9±0.31
	II	2.8±0.31	0.3±0.48	0.8±0.73	2.8±0.42	2.8±0.31	2.9±0.31
	III	2.4±0.52	0.1±0.31	1.0±0.73	2.5±0.52	2.4±0.52	2.6±0.53
Day 28	I	3±0	0.0±0	0.0±0	1.9±0.73	1.8±0.91	3.0±0
	II	2.9±0.31	0.0±0	0.3±0.94	1.8±0.91	2.0±1.15	3.0±0
	III	2.7±0.48	0.1±0.31	0.6±0.52	1.9±0.73	2.2±0.78	2.8±0.31

Group I=Animals treated with PRP, Group II=Animals treated with asiaticoside at 2%, Group III=Animals treated with saline solution (NaCl 9%),

* =Denote a significant difference (p<0.05). PMNL=Polymorphonuclear leukocyte, SD=Standard-deviation, PRP=Platelet-rich plasma

At the 3^rd^ day of the healing evolution, epithelialization was observed on the inner wall of the free edge of wounds treated with PRP (Figure-4a), whereas in the wounds of the group treated with asiaticoside, it was less important (Figure-4b). No epithelialization was observed in the saline-treated wounds, with wounds appearing covered with blood coagulum (Figure-4c).

Regarding the semi-quantitative evaluation of epithelialization, our results revealed that a significant difference (p<0.05) recorded at the 21^st^ day of healing between the group of wounds treated with PRP and the group of control wounds: (3.0±0) against (2.4±0.52), respectively. Concerning inflammatory cells (polymorphonuclear leukocyte [PMNL]), our histopathological examination revealed a massive infiltrate at the edges of the PRP-treated wounds compared with the wounds of the other groups (Figure-5a). In addition, the semi-quantitative evaluation revealed a significant difference (p<0.05), observed in favor of PMNL (wounds of Group I: 2.4±0.48, compared with wounds of Groups II and III: 1.9±0.73 and 1.5±0.7, respectively). For the other parameters, no histopathological changes were observed, and no organization of newly formed collagen was evident in the different groups.

At day 7, the histopathological examination revealed a certain accentuation of the degree of epithelialization observed in different wounds. PRP-treated wound edge surfaces appeared (Figure-4d), entirely covered by epithelial tissue compared to other control wounds (Figure-4e and f). However, no connection of the edges of wounds was observed in the different groups. In addition, the semi-quantitative evaluation of epithelialization revealed no significant difference. Concerning the collagen, his organization seemed much denser and organized at the level of wounds treated with PRP (Figure-4d) in comparison with other wounds. For other parameters, semi-quantitative evaluation revealed significant variations in fibroblasts and macrophages (Table-3). Regarding fibroblasts (wounds of animals of Group I: 2.1±0.73 against the wounds of animals of Groups II and III: 1.7±0.48 and 1.6±0.51 respectively), for macrophages (Group I wounds: 1.2±0.42 compared to Group III wounds: 1.6±0.91, respectively).

At day 14, histopathological examination showed a cardinal change in wound healing, characterized by the union of wound margins in the different groups (Figure-4), almost complete with better granulation tissue in wounds treated with PRP (Figure-4g). In addition, the fibroblast variation study revealed a significant difference (p<0.05) in favor of wounds of Group I: 2.7±0.48 against wounds of Group III: 2.4±0.51.

At day 21, complete epithelialization was observed in wounds treated with PRP, with fully joined tissues. The new collagen bundles were densely filled the site of the wound treated with PRP (Figure-4j). In the other groups, we noted the continuation of the rehandling of the epithelialization and the reorganization of the collagen (Figure 4k and l). The semi-quantitative evaluation revealed a highly significant difference in favor of PRP-treated wounds fibroblasts: (3.0±0 compared with wounds of Group III: 2.4±0.52).

At day 28, histopathological examination showed wound healing in the different groups. Perfect healing was observed in wounds of Group I, characterized by a reorganization of collagen with keratinization of the skin and reappearance of hair follicles in the site of wound (Figure-4m). Poor scarring was observed in the other groups (advanced fibrosis in wounds treated by asiaticoside, poor delayed healing in control wounds). For the other parameters, our study was showed that the number of newly formed capillaries increased considerably during the first 2 weeks following the injury and that it only decreased after the 3^rd^ week of evolution. On the contrary, with regard to the newly formed collagen, its proportion was continuously growing.

## Discussion

Nowadays, the use of PRP offers a new multidisciplinary approach in full evaluation whether in human or veterinary medicine [1]. In this context, several widely controversial studies have been published to evaluate their biological effects of tissue regeneration, through platelets and growth factors that they released at the site of injury [3,4]. Its autologous character gives it, moreover, excellent biocompatibility and biodegradability, while avoiding the risk of transmission of pathogens. In humans and animals, numerous publications have been reported on its use in a wide variety of medical fields (osteoarticular, maxillofacial, neurosurgery, skin scarring, plastic surgery, etc.) [12,13]. However, in sheep, there is little research on the evaluation of its effect on tissue scarring, particularly cutaneous, where most researchers have focused on osteoarticular investigation [13]. In addition, some authors have reported that the prevalence of skin lesion complications in ruminants, have a tendency to increase dramatically (infections such as mastitis and panaris, parasitic skin infestations with different forms of gal and burns in case of fire, etc.) [14]. According to Chicharro-Alcántara *et al*. [12] scarring can have a biomedical and economic impact that is sometimes feared. Therefore, the need to establish new strategies, such as regenerative therapies based on the PRP, remains a challenge. This research aims to examine the effect of PRP, with the hypothesis that the latter stimulates epithelialization and keratinization, accelerates contraction and promotes collagen remodeling in different tissues. We performed a comparison of cutaneous healing using three different treatments (PRP, asiaticoside, and saline) on wounds of complete excision of the skin in sheep.

In our study, the inspection of skin excision sites during the first days after injury of the skin revealed no complication or apparent signs of infection. This could be due to the excellent care of the wounds and the small number of animals used. Our results are in accord with those reported from numerous studies. Smets *et al*. [2] suggested that the biological nature of PRP could significantly affect the healing process especially when the PRP is prepared by simple centrifugation. The latter may contain variable amounts of cells of the white line, responsible for the degradation of the extracellular matrix by releasing pro-inflammatory factors [4,15]. Indeed, the results obtained from this recent study, confirm that our PRP was practically free of all cells of the white line (Figure-1b). According to Kaux *et al*. [4], the use of a double centrifugation regime is unavoidable, because this method also makes it possible to avoid-free radicals derived from residual red blood cells, thus being able to create infectious foci harmful to tissue structures. Concerning the number of platelets obtained using this protocol, our results are within very good concentration limits established by Arora *et al*. [16] and Whitman *et al*. [17]. These authors stated that the ideal platelet concentration for PRP to present stimulatory properties would be 2-3 folds higher. Regarding the choice of anticoagulant, the repeated observations of the different smears performed (Figures-1a and b), confirmed the effectiveness of dextrose during the preparation of PRP in sheep. Lei *et al*. [18] reported that dextrose (ACD) was the appropriate anticoagulant for PRP preparation, and the quality of PRP was closely related to the type of anticoagulant used.

The morphometric study of the percentage of contraction of wounds has shown that there is remarkable progress in the skin healing process for animals of Group I treated with PRP. At the 7^th^ day of evolution, a significant difference was observed between wounds of Group I, and wounds of Groups II and III. This difference becomes glaring and highly significant at the 14^th^ day of healing progress. Our results are similar to those reported by some authors. Indeed, Al-Bayati *et al*. [8] confirmed that the subcutaneous infiltration of PRP accelerates the contraction of wounds in the goat from the 1^st^ post-operative week. Some authors [3,19] reported that this acceleration of contraction is directly related to the potential effects of therapeutic agents presented by growth factors accumulated in the platelet granules. These factors include vascular endothelial factor, which promotes angiogenesis, platelet-d-rived growth factor, and transforming growth factor-β. These factors contributed in particular to the migration of leucocytes to the site of the wound [20,21], as well as to the proliferation of fibroblasts in myofibroblasts and to the synthesis of the constituents of the extracellular matrix. Other authors reported that contraction progress related to both myofibroblasts and fibroblasts that can organize connective tissue with the early consolidation of collagen by pulling the skin inward [19,22].

Semi-quantitative evaluation of histopathological scores obtained revealed significant differences for a certain number of healing parameters. Indeed, our results confirm that the topical application of PRP allowed a good reepithelialization (Figure-4m), with earlier neovascularization, followed by a more elaborate collagen reorganization. Alishahi *et al*. [23] reported that skin infiltration of PRP accelerated wound healing in dogs and allows having a more elaborate granulation tissue.

At the 3^rd^ day of healing, our results are encouraging, testifying the biological effectiveness of PRP, the latter capable potentially accelerating of healing process by massive recruitment of nuclear polymorphs (Figure-5a). In addition, this new technology could also increase the rate of angiogenesis. Our results are in agreement with those reported in many studies, Ostvar *et al*. [6] reported that the use of PRP increases the rate of new loops of capillaries observed in skin wounds in rabbits. Darré *et al*. [22] suggested that this neovascularization during skin wounds favors the arrival of inflammatory cells with a function of debridement of the lesional focus in association with macrophages [24]. However, repeated histopathological observations of biopsies conducted from wounds treated with PRP, revealed that the number of macrophages was less important significant compared with the wounds of the other groups (Figure-5c). This confirms the finding already established by Kaux *et al*. [4], who suggested that the use of PRP by tendon infiltration delimited chronic inflammation by a reduction of the number of macrophages.

Concerning the study of fibroblast variation, our results revealed significant differences recorded at the 7^th^ and 14^th^ day of healing, in favor of wounds of animals of Group I. This could be explained by some authors [20,21], to the associated effects of certain PRP growth factors that can promote fibroblast differentiation and collagen formation. In addition, the careful observation of the various histopathological aspects of the micrograph of Figure-6, allowed us to think that this preparation (PRP) could contribute in the process of remodeling and reworking of collage. Indeed, Kaux *et al*. [4] reported in another similar study in humans that the level of collagen in the group treated by tendon infiltration of PRP increased significantly at the 5^th^ day of healing.

However, the exact mechanism of PRP action in collagen remodeling is still unclear. Ibrahim *et al*. [9] have suggested that collagen remodeling is likely to be affected by metalloproteinases, especially MMP-1 and MMP-3 proteins that can digest the various structural components of ECM. In addition, the induction of MMP-1 in the skin can facilitate the removal of damaged collagen fragments in the tissue of the dermal matrix, thus providing a better basis for the deposition of new collagen necessary for the improvement of wounds.

Finally, concerning the reconstruction of hair follicles, the results of this present study have showed that there is some reconstitution of the dermal papilla cells, observed in the site of healing for wounds treated by PRP. Our results are in agreement with those established by Ferdousy *et al*. [25]. These authors reported in a similar study in the goat that intradermal injection of PRP into skin wounds greatly improved healing with the appearance of hair follicles. This could be explained by Wang *et al*. [26], with the potential effects of PRP specially growth factors, which significantly increases the level of Pal (alkaline phosphatase) and versican (chondroitin sulfate proteoglycan). These can promote the proliferation of human hair dermal papilla cells. In addition, Miao *et al*. [27] have suggested that the previously mentioned growth factors may induce hair follicle reconstitution; however, the exact tests on its effectiveness are limited.

## Conclusion

Wound healing is a complex process, resulting from the interaction between a large number of cell types, extracellular matrix proteins, and mediators such as cytokines and growth factors. In this study, we evaluated the therapeutic efficacy of PRP obtained by a double centrifugation protocol, on cutaneous wound healing induced by complete excision of the skin in sheep. Our results showed that the use of this topical in sheep, reduced inflammation during the first 3 days of healing, reduced the contraction surface of wounds from the 1^st^ week post-surgical and promotes epithelialization in 3 weeks of healing. All of these current findings should be accepted as preliminary. Finally, to arrive at full scientific validity, it seems necessary to consider other methods of investigation, using advanced techniques to clarify in an exact way the biological effects of the PRP.

## Authors’ Contributions

DB and BO have contributed equally in this research. Both authors read and approved the final manuscript.
